# Health policy mapping and system gaps impeding the implementation of reproductive, maternal, neonatal, child, and adolescent health programs in South Sudan: a scoping review

**DOI:** 10.1186/s13031-020-00258-0

**Published:** 2020-04-14

**Authors:** Loubna Belaid, Pontius Bayo, Lynette Kamau, Eva Nakimuli, Elijo Omoro, Robert Lobor, Baba Samson, Alexander Dimiti

**Affiliations:** 1 Family Medicine Department, McGill University, 5858 Chemin de la Côte des Neiges, Montréal, Québec Canada; 2 Ste Mary’s Lacor Hospital, Gulu, Uganda; 3 African population and health research center, Nairobi, Kenya; 4 Partners in Population and Development Africa Regional Office, Kampala, Uganda; 5 Torit State Ministry of Health, Juba, South Sudan; 6 WHO, South Sudan Country Office, Juba, South Sudan; 7 Ministry of Health, Juba, South Sudan; 8 Department of Reproductive of Health, Ministry of Health, Juba, South Sudan

**Keywords:** South Sudan, Scoping review, Reproductive maternal newborn child health, Policies, Programs, Health system gaps, Fragile states

## Abstract

**Background:**

Pregnant women, neonates, children, and adolescents are at higher risk of dying in fragile and conflict-affected settings. Strengthening the healthcare system is a key strategy for the implementation of effective policies and ultimately the improvement of health outcomes. South Sudan is a fragile country that faces challenges in implementing its reproductive, maternal, neonatal, child, and adolescent health (RMNCAH) policies. In this paper, we map the key RMNCAH policies and describe the current status of the WHO health system building blocks that impede the implementation of RMNCAH policies in South Sudan.

**Methods:**

We conducted a scoping review (39 documents) and individual interviews (*n* = 8) with staff from the national Ministry of Health (MoH) and implementing partners. We organized a workshop to discuss and validate the findings with the MoH and implementing partner staff. We synthesized and analyzed the data according to the WHO health system building blocks.

**Results:**

The significant number of policies and healthcare strategic plans focused on pregnant women, neonates, children, and adolescents evidence the political will of the MoH to improve the health of members of these categories of the population. The gap in the implementation of policies is mainly due to the weaknesses identified in different health system building blocks. A critical shortage of human resources across the blocks and levels of the health system, a lack of medicines and supplies, and low national funding are the main identified bottlenecks. The upstream factors explaining these bottlenecks are the 2012 suspension of oil production, ongoing conflict, weak governance, a lack of accountability, and a low human resource capacity. The combined effects of all these factors have led to poor-quality provision and thus a low use of RMNCAH services.

**Conclusion:**

The implementation of RMNCAH policies should be accomplished through innovative and challenging approaches to building the capacities of the MoH, establishing governance and accountability mechanisms, and increasing the health budget of the national government.

## Background

It has been estimated that 2 billion people live in areas affected by fragility, conflict and violence [1]. According to the United Nation High Commissioner for Refugees, the number of forcibly displaced people has nearly doubled in the past two decades (from 33.9 million in 1997 to 65.6 million in 2016) [2]. This number is the highest it has been since World War II. It has been reported that half of refugees are children [2].

Women, adolescents, newborns, and children are at higher risk of dying in fragile and conflict-affected settings [3]. **In 2015, 61% of maternal deaths occurred in 35 countries affected by emergency crises or fragile conditions** [3]. The 10 countries with the highest under-five mortality rates (U5MRs) are all in sub-Saharan Africa and have U5MRs above 90 per 1000 live births. Nine of these countries are fragile and conflict-affected settings (Nigeria, Angola, DR Congo, Benin, Central African Republic, Equatorial Guinea, Somalia, Chad, Mali, and Sierra Leone) [4].

Conflicts not only contribute to increased mortality and morbidity rates but also seriously affect already vulnerable healthcare systems through the destruction of infrastructure, the flight of healthcare workers, and the interruption of the delivery of drugs and medical supplies [5–7].

Humanitarian agencies provide quick emergency responses during and in the aftermath of conflicts. Their responses are often characterized by structured vertical programs ranging from mass immunization, nutrition, reproductive health, emergency surgery, and mental health services [8, 9]. Humanitarian agencies have achieved some success in reducing maternal, neonatal, and child morbidity and mortality rates in very difficult conditions [10, 11]. However, as conflicts increase and their impact becomes globalized, the global community is shifting humanitarian responses so that more important investments are made to strengthen healthcare systems, increase the resilience of populations, and reduce risk and all forms of vulnerability [12–15].

Given the magnitude of the problem and this new shift in the humanitarian response, there is limited evidence on how to strengthen healthcare systems in fragile and conflict-affected settings to implement effective reproductive, maternal, newborn, child, and adolescent health (RMNCAH) programs and policies [5, 12, 16, 17].

The WHO health system building blocks framework has the potential to highlight broader healthcare systems challenges [18]. In this paper, we map the key RMNCAH policies, describe the current status of the WHO health system building blocks, and highlight the key challenges that impede the implementation of RMNCAH policies and programs in South Sudan.

### Context

Sudan went through long periods of internal conflicts due to the First Sudanese Civil War (1955–1972) and the Second Sudanese Civil War (1983–2005). The comprehensive peace agreement signed between the Government of Sudan and the Sudan People’s Liberation Movement/Army in 2005 ended the longest African conflict [19]. Six years later, South Sudan gained its independence (2011). At its independence, South Sudan, the youngest nation of the world, faced widespread poverty, almost nonexistent basic infrastructure, and weak government institutions.

**Fig. 1:**
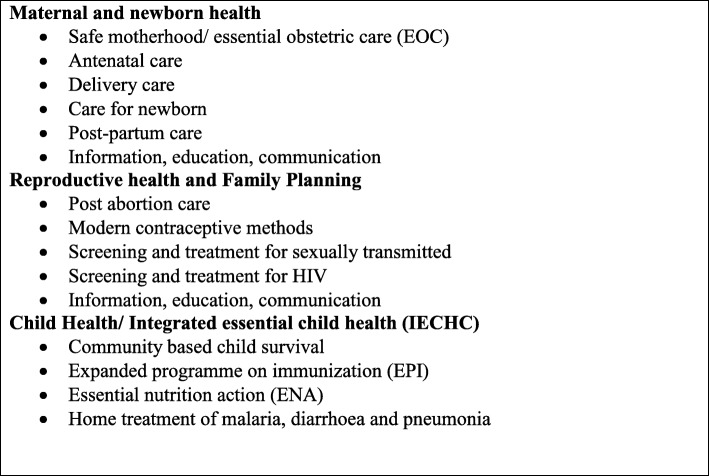
Maternal, newborn, reproductive, and child health services in the Basic Package of Health and Nutrition Services in South Sudan

In 2011, the Ministry of Health (MoH) identified and prioritized a list of basic health services, known as the Basic Package of Health and Nutrition Services (BPHNS), that should be affordable and accessible to the majority of the population at the primary and secondary healthcare levels. The BPHNS covers curative, promotive, preventive, and managerial activities. It is the cornerstone of the National Health Policy (2016–2026) and health strategic plans (Fig. 1).

It is financed by a combination of domestic revenues and aid from major bilateral and multilateral donor agencies such as the South Sudan Health Pooled Fund (HPF) and the World Bank (WB). The South Sudan HPF is a multi-donor funding mechanism that currently includes six donors: the United Kingdom, Canada, the European Union, Sweden, the United States, and GAVI. This funding mechanism operates in 23 geographic areas in eight of the 10 former states by contracting NGOs. They use MoH facilities and health staff [20].

The HPF is currently in its third phase, which will run until July 2023. This phase focuses on two programs: the provision healthcare services at the health facility level and expanded community health services based on the Boma Health Initiative (BHI) structures [20].

**Fig. 2:**
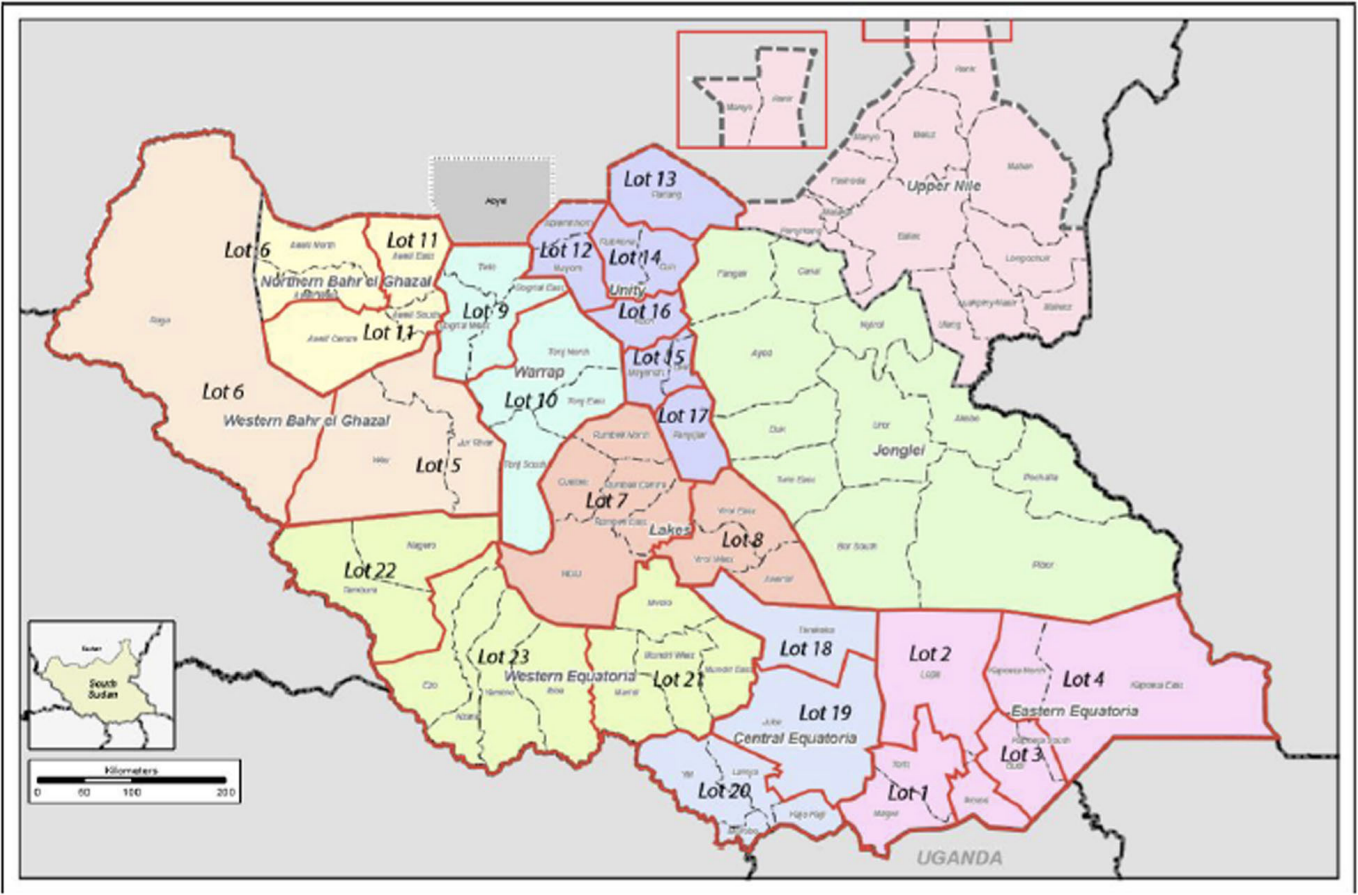
Health pool fund geographic distribution by lot, South Sudan (2018), Source*: Evaluation HHP2, 2018

The WB works through UNICEF to provide health services in the two former states where HPF does not operate, Jonglei and Upper Nile. The WB program also contracts with other NGOs and county health departments as implementing partners (Fig. 2) [20].

## Methods

We conducted a scoping review, in-depth interviews, and a workshop.

### Scoping review

We used the Arksey O’Malley and Levac frameworks [21, 22] to conduct a scoping review of policy documents on RMNCAH in South Sudan. The framework for the review was based on six steps.

#### Step 1: identification of the research questions

The research questions were as follows: (1) What are the policy documents used by the MoH to guide policies and programs for improving RMNCAH in South Sudan? (2) What are the health system gaps impeding the implementation of RMNCAH policies and programs?

#### Step 2: identification of relevant documents

We focused on documents that had been published by the government of South Sudan and peer-reviewed publications on South Sudan published between 2011 and January 08, 2019.

We used several strategies to identify relevant documents. For peer-reviewed publications, we searched Medline (global health), PubMed, Web of Science, and Google Scholar in September 2018 using the following MeSH terms and Boolean operators (AND, OR): [“Health policy” or “health system” or “health program” or “health services”] and [“guidelines” or “strategies” or “plans”] and [“South Sudan”].

For policy documents, we explored several governmental and organizational websites. We checked the reference lists of the documents and relied on our network in South Sudan to access documents published by the MoH.

#### Step 3: screening and selection of relevant documents

Two reviewers (LB and PB) independently screened and selected the relevant documents. We selected documents focused on RMNCAH in South Sudan. We restricted the language to English and French. For peer-reviewed publications to be included, the documents had to describe empirical data on health system building blocks that were collected using any study design (quantitative, qualitative, or mixed). We excluded conceptual commentaries and clinical and biomedical papers. For policy documents to be included, the document needed to cover topics related to RMNCAH.

#### Step 4: charting of the data

We developed a data charting form to extract the data from each selected document. We extracted descriptive data such as the title, type of document (peer-reviewed publication, health policy, health strategic plan, or health system assessment), area of RMNCAH, study design, use of evidence, year of publication, and population. We used the WHO health system building blocks framework to organize and analyze the data [23].

#### Step 5: collation, summary and reporting of the results

We used tables to describe synthesized descriptive and analytical data [24]. We identified the key themes emerging from the documents for each health system building block.

To assess the quality of the studies, we used an intermediate approach that is recommended for reviews that combine quantitative, qualitative, and mixed methods studies [25]. In scoping reviews, the included studies are neither hierarchized according to their study designs nor attributed a weight for their data [21]. Nonetheless, Table 2 shows the robustness of the study design of the included studies.

#### Step 6: consultation – workshop

The workshop allowed us to discuss, disseminate, and validate the findings with the key stakeholders in charge of the formulation, implementation, evaluation, and monitoring of RMNCAH policies and programs in South Sudan. The workshop was held in Juba, the capital city, on December 3–4, 2018, and included directorates from the MoH and international and national partners [*n* = 25 participants).

### Individual interviews

We conducted eight in-depth interviews with the directors of the MoH and implementing partners in Juba using an interview guide. The participants who were interviewed attended the workshop. Each interview lasted between 30 min and 2 hours. The first author (LB) conducted the interviews in English. We did not record the interviews to encourage participants to express themselves freely. We took systematic notes for each individual interview.

In the interviews, we explored the priorities of the government regarding RMNCAH programs and policies, the health system gaps impeding the implementation of RMNCAH programs/policies, the use of evidence, and the cross-cutting dimensions (gender, community participation, and equity) in the formulation of RMNCAH policies and programs. This study was part of a larger study on health policies in South Sudan. However, in this paper, we describe only the results related to the key RMNCAH policies and the current status of the WHO health system building blocks.

We used thematic analysis with a mixed approach (deductive and inductive) to analyze the interviews [24]. The deductive themes were identified from the WHO health system conceptual framework, and the inductive themes were generated from the empirical data to allow the emergence of unexpected outcomes, mechanisms, and challenges related to the research objectives. The triangulation of the data generated by the scoping review, the in-depth interviews, and the workshop contributed to enhancing the internal validity of this study [47]. We used the Preferred Reporting Items for Systematic Reviews and Meta-analyses Extension For Scoping Reviews (PRISMA-ScR).

### Ethical considerations

We obtained ethical approval and consent for publication for this study from the ethical committee of the MoH, Republic of South Sudan (MoH/ ERB 472018). We obtained informed consent from the participants and maintained confidentiality of all the data obtained.

## Results

### Description of the identified documents

The review included 39 documents. In total, 16 documents were policy documents (national health policies, health strategic plans, guidelines, health assessments, and health situation analyses), and 23 were peer-reviewed publications. The characteristics of the documents are detailed in Tables 1 and 2.

**Table 1:** Description of policy documents included

Number	Title	Year of publication	Type of documents	RMNCAH areas	Health system’s building blocks
1	Basic package of health and nutrition services in primary health care	2011	Implementation guideline	Integrated	NA
2	The Family Planning policy	2013	Health policy	Family Planning	All
3	The National Health Policy (2016-2025)	2016	Health policy	Integrated	NA
4	The National health strategic plan (2016-2020)	2016	Health strategic plan	Integrated	All
5	South Sudan adolescence sexual and reproductive health strategic plan	2018	Health Strategic plan	Adolescent’s Sexual & reproductive health	All
6	The Community health system in South Sudan: The Boma Initiative	2016	Implementation guideline	Integrated	NA
	The National	2018	Health policy	Integrated	All
7	Reproductive health strategy (2018-2022)				
8	Health Strategic Plan	2017	Health strategic plan	Integrated	All
9	South Sudan National Emergency Obstetric and newborn care needs assessment	2014	Nationwide health assessment	Maternal Newborn	All
10	Rapid facility survey	2013	Nationwide health assessment	NA	NA
11	Rapid assessment of the status of RMNACH and nutrition services in South Sudan	2014	Report	Integrated	All
12	Health situation analysis for the national health policy update	2014	Report	Integrated	All
13	Every newborn action plan	2018	Health strategic plan	Newborn	All
14	Midwifery training policy	2018	Implementation guideline	Maternal and newborn	Human Resources
15	Maternal infant and young child nutrition (2017-2025) strategy	2017	Health strategic plan	Pregnant women, newborn and children	NA
16	Maternal infant and young child nutrition (2017-2025) guidelines	2017	Implementation guideline	Pregnant women, newborn and children	NA

**Table 2:** Description of peer -reviewed publications

Authors & Date	Study design & data collection	Settings	Population & Sample size	RMNCAH	Health system ‘s building blocks assessed
Berendes and al (2014) [26]	health facility assessment Cross sectional survey Infrastructure Material and supplies Health workers Patient records	Nationwide	14-16 Health facilities in each 10 States (n=156 randomly selected facilities)	Children consultation Antenatal care	- Service delivery (quality of care, accessibility) - Medicines and supplies - Information system - Health workforce (performance)
Bayo and al. (2018) [27]	Retrospective -cross sectional study	Torit County	2466 patient’s admission files	Obstetric care (emergency obstetric complications)	Service delivery
Elmusharaf and al. (2017) [28]	Participatory Ethnographic evaluation and research In depth interview Workshop	Renk County, Northern Upper Nile State	14 women selected from villages of Renk County	Family planning (family size)	Community level
Kane and al. (2016) [29]	Qualitative exploratory study FGD (n=5) Individual interviews (n=44)	Wau County, State of Western Bahr el Ghazal (Fertit community: rural and urban)	Community members (male and female) Traditional healers Traditional birth attendants Health facility personnel State SRH managers NGO representation	Family planning (social norms shaping birth spacings, childbearing Marriage)	Community level
Kane and al. (2018) [30]	Qualitative exploratory study FGD (n=5) Interviews (n=44)	Same as Kane and al. (2016)	Same as Kane and al. (2016)	Maternal health (perception on the use of services for childbirth)	Service delivery (facility-based services)
Lawry and al. (2017) [31]	Cross sectional study Randomized household survey quantitative qualitative interviews (n=72)	Gogrial West,Warrap State	Pregnant women or had children less than 5 years of age (n=860) Men (=144) Qualitative interviews (n=72)	RMNCH Contraception Facility based-deliveries Antenatal care Malaria (mosquito nets) DPT3 immunization Gender based violence	Service delivery Community level (knowledge on danger signs of maternal newborn and child health, perceptions of gender norms related to RMNCH health)
Mugo and al. (2016) [32]	Cross sectional 2010 South Sudan household survey	National	2767 of mothers who gave birth within 2 years prior the survey and delivered their babies at home	Maternal health (deliveries)	Service delivery
Mugo and al. (2015) (ANC) [33]	Analysis of 2010 South Sudan household survey (a nationally representative, stratified, cluster sample survey)	National	3504 women aged 15-49 years who had given birth with 2 years preceding the survey	Antenatal care	Service delivery
Mugo and al. (2015) [34]	Literature review on South Sudan	National	NA	MNCH	Health workforce Governance/ Leadership Service delivery Medicines/ supplies
Mugo and al. (2018) [35]	Qualitative study (in depth interviews)	Juba County (central Equatoria state)	Women with children aged less than 3 months, 10 in each type of health care facility who had given birth either at home or in a health facility(n=30), husbands (n=15)	Maternal and child health	Service delivery
Mugo and al. (2018) [36]	Analysis of 2010 South Sudan household survey	National	8125 singleton, live birth, under-five children born in South Sudan within 5 years prior to the 2010 South Sudan Household Survey	Child health	Service delivery
Palmer and al. (2016) [38]	Ethnographic analysis of public health interventions (document analysis, observation) key informant interviews (n=54)	Juba	Health managers from the MOH, Ministry of Gender, Local and international NGO’s staff and UN agencies nurse and local women’s organization Journalists	Family planning Abortion	NA
Sami and al. (2018) [39]	Cross sectional descriptive study of facility -based deliveries (clinical observation, exit interviews, health facility assessment, direct observation of midwife time use)	displacement camps: Maban Gendrassa Kaya, Juba, and Malakal	Mother-newborn pairs who sought services and birth attendants who provided delivery services between April and June 2016 in 5 health facilities	Maternal health Newborn health	Service delivery: Quality of care for mothers and newborns
Scott and al. (2013) [40]	Community-based participatory research Using quote sampling	Aweil (Northern Bahr El Ghazal) Wau (Westen Bahr El Ghazal) Lainya, Morobo, Ronyi (Central Equatoria) Malakal (Upper Nile State)	N= 680 respondents, n=352 female, n= 326 male	Gender norms regarding sexuality and reproductive health	Community level
Izudi and al (2017) [41]	cross sectional study (survey with random sampling)	Mundri East County (Westen Bahr El Ghazal)	13 (one regional referral hospital, 2 county hospitals, 10 PHCC) postpartum mothers that had live births, were 15–49 years old, 8–14 days post-delivery and that attended PNC clinics (to receive immunization, contraception and growth monitoring services)	Postnatal care	Service delivery
Kane and al. (2016) [29]	Qualitative descriptive study FGD (n=5) Interviews (n=44)	Wau County, State of Western Bahr el Ghazal	Same as kane and al. 2016, Kane and al. 2018	Gender norms related to reproductive health	Community level
Sami and al. (2017) [42]	Cross sectional study based on self -administered questionnaires and in-depth interviews	IDP (Sami and col. 2018)	Health providers Community health workers Traditional Birth Attendants	Maternal health (childbirth, post -partum) newborn’s health	Quality of care (knowledge and practice of care for mothers and newborns)
Wilunda and al. (2016) [43]	Qualitative study FGD (n=14) Key informant interviews (n=12)	Rumbek North County	Women (n=169), men (n=45) community leaders, health providers, and the staff of the County Health Department	ANC	Service delivery
Jones and al. (2015) [44]	Qualitative study	Nationwide	International health staff (technical advisor, consultant, senior managers, supervisors) from international, local organizations, health providers (n=20)	Primary health care (MNCH)	Six building’s blocks
Myake and al. (2017) [45]	Scoping review	South Sudan	N=2 papers on South Sudan	RMNCH	Human resources (community midwifery)
Sami and al. (2018) [39]	Case study using mixed methods (FGD (n=19), in-depth interviews (n=7) observation of health facility readiness, documents)	IDPs, Hospital (n=1) PHCC (n=4) CH programs (n=4)	Health workers (n=43) CHW (n=61) Program managers (n=7)	Newborn care	All 6 building blocks
Kane and al. (2018) [30]	Exploratory qualitative study (FGD (n=4), in-depth interviews (n=44)	Wau county	Married women, 18- 35 years of old; unmarried women, 18- 35 years of old; men older than 35 years; men younger than 35 years health workers	Gender norms related to reproductive health	Community level
Kane and al. (2019) [46]	Exploratory qualitative study (individual interviews)	Wau county	Teenage females and males (in school, out school, with child, no child) Parents	Reproductive health (teenage pregnancy, views of childbearing)	Community level

### RMNCAH policy mapping

Since its independence, South Sudan has placed a strong emphasis on improving RMNCAH outcomes through the formulation and implementation of its health policies and strategic health plans, as shown in Table 1.

The BPHNS is a list of preventive and curative packages of services that should be provided at the primary and secondary healthcare levels. The MoH produced specific health strategic plans for specific components of reproductive health (family planning) and for targeted populations (adolescents and newborns) from 2012 to 2016.

*“These strategic plans provided more details on strategies and activities than the broader health sector strategic plan”* (female, program specialist, implementing partner #1*).*

The main issue faced by the MoH is the effective implementation of these RMNCAH policies:

*“We have acknowledged mothers’, adolescents’ and children’s health problems, but it is the implementation of the programs which is the biggest challenge”* (male, technical officer, implementing partner #2*).*

### Health system gaps

#### Service delivery

##### Low coverage of RMNCAH services

The coverage for most RMNCAH services remains low. For instance, the rate of assisted facility-based deliveries was estimated to be 21% according to 2010 Demographic Health Survey data (DHS) [48]. The rates of fourth antenatal care visits and postnatal care visits in urban and rural areas were estimated to be 15 and 8% and 13 and 9%, respectively. The DPT vaccination coverage rates for children aged 12–23 months were 25, 20, and 13% for the first, second, and third doses, respectively. Only 2.6% of children had all nine recommended vaccinations, [48] and only one in five children aged 1 year or less were immunized against measles [49]. One-quarter of children under five were found to be stunted due to inadequate nutrition [49].

A study that assessed the factors associated with under-five mortality indicated that on average, approximately 50% of children under five had no access to evidence-based interventions, such as insecticide-treated mosquito nets (34%), improved sources of drinking water (69%), improved sanitation facilities (7%), rehydration treatment for diarrhea (49%), antibiotic treatment for pneumonia (33%), and childhood immunizations (6%) [37]. A randomized household quantitative study conducted in Warrap State reported that most women (90.8%) and men (96.6%) did not want contraception. Only 1.2% of women aged 15–49 had their needs met for family planning [31]. These poor health indicators are related to the low access to healthcare services for women, newborns, and children [31–33, 35, 43]. We did not find data on adolescent health due to a lack of disaggregated data (Tables 3 and 4).

**Table 3:** Selected RMNCH coverage indicators

Indicators	Value	Source of information	Year of estimate
Contraceptive prevalence rate	3%	UNFPA	2015
Unmet need for contraception	24%	UNFPA	2015
Proportion of mother receiving at least 4 ANC	17%	WHO	
DPT3 coverage (12-24 months of age) before 12 months	45.1%	South Sudan coverage Survey	2012
Measles coverage (12-24 months of age)	45%	South Sudan coverage Survey	2012
Proportion of children who slept under an ITN in the previous night	25%	UNICEF	2015
Proportion of infants under 6 months exclusively breastfed	45%	UNICEF	2015
Proportion of HIV+ mothers who received ART prophylaxis	18%	HIV/AIDS Commission Report	2014

**Table 4:** Selected RMNCAH Health Status indicators

Indicator	Value	Source of information	Year of estimate
Maternal mortality ratio	789/100,000 live birth	UN-interagency Estimates	2015
Neonatal mortality rate	39/1000 live birth	UN-interagency Estimates	2015
Infant mortality rate	60/1000 live birth	UN-interagency Estimates	2015
Under-five mortality rate	98(M), 87(F) / 1000 live birth	UN-interagency Estimates	2015
Total Fertility Rate	4.9	WHO/SSD statistical profile	2013
Adolescent Pregnancy Rate	31%	SSHHS	2010
HIV prevalence rates among pregnant women	2.5%	SS Spectrum Estimates	2015

##### Several barriers to accessing RMNCAH services

Multiple quantitative and qualitative studies explored the barriers to accessing maternal healthcare services [27, 30–32, 41, 43]. The maternal health services assessed in the studies were antenatal care, facility-based deliveries, and postnatal care. Only one study reported data on access to healthcare services for children (immunization and curative care for respiratory infection, malnutrition, and diarrhea) [31]. We did not find studies on access to health services for adolescents.

We identified individual and structural factors that negatively influence access to maternal healthcare services. At the individual level, the level of education and knowledge of newborn danger signs are associated with the nonuse of antenatal care [33]. A mixed method study conducted in Gogrial West and Warrap States reported limited recognition of maternal and newborn danger signs as well as childhood illnesses [31].

The structural factors identified in studies using qualitative data collection methods such as focus group discussions and individual interviews included distance, a lack of means of transport, service costs, sociocultural factors related to gender norms, and insecurity [35, 43].

*“Meen hospital [primary healthcare unit (PHCU)] and Maper hospital [primary healthcare center (PHCC)] are very far from us. We are actually in the middle between Rumbek and Maper hospitals. If you want to go to hospital, you can spend one day to reach there”* [43]*.*

*“What I dislike about the hospital is that after delivery, the mother is asked to pay money, but we don’t have money; we just go there to get help”* [43]*.*

*“I only attended one antenatal care service during my pregnancy. Everything here (at the hospital) is at a cost, and we are suffering financially. The little (money) we have is for buying some food”* [35]*.*

*“The husband is the one who decides where a woman should give birth. Even if a woman has decided to deliver in the hospital, the husband will say, ‘No, you are just going to roam there; you must deliver here. Whom will you leave your children with if you decide to go and deliver in the hospital?’ Our husbands decide where we should deliver”* [43]*.*

*“Our place is also in the middle of enemies who frequently attack us. Some of us fear that if we go to deliver in the hospital and the enemy comes to attack in our absence, there will be nobody to lead our children to a hiding place”* [43]*.*

The low use of family planning services was found to be influenced by social and cultural norms [28, 29, 38]. A study conducted in Renk County based on a participatory ethnographic approach indicated that the participants experienced pressure to increase the size of their families. The determinants influencing family size were cultural practices, clan lineage, the loss of family members, and high rates of child mortality. The cultural practices included the belief that marriage is incomplete when no child is conceived, fears associated with infertility, and the lack of social status for women without children [46].

*"If you are married and already living with your husband and do not have a child, the.*

*husband can leave you and tell you to go back to your family*" [30]*.*

*“His relatives will come and argue about why you are not getting pregnant … the man’s relatives will complain, ‘Why is this woman brought and eating our food for free if she is not going to deliver children?’”* [30].

##### Perceptions of poor quality of care

The poor quality of care perceived by the population was also reported as a major barrier to access to healthcare services [30, 35]. Poor quality of care was described as a lack of medicines, supplies, and skilled staff and poor attitudes towards patients among health staff [35, 43].

*“Most of the time, there is not enough medicine, and after long waiting times, we are asked to come back next day. It’s hard for me since I am unwell and too weak to do that. If I had money, I would have bought these medications from the private pharmacy”* [35]*.*

A national cross-sectional survey of 156 randomly selected health facilities in 10 states using two-stage lot quality assurance sampling reported the general poor quality of care in the 10 states. In the study, all of the health facilities failed to reach the 80% targets for 14 of 19 indicators of quality of care, and few or no facilities were categorized as acceptable regarding adequate utilization by the population for sick-child consultations (12%), staffing (16%), the availability of infection control supplies (3%), and the presence of all child care guidelines (0%) [26].

The MoH conducted a nationwide assessment of emergency obstetric and newborn care services using a cross-sectional facility-based survey. The report indicated that of 50 hospitals (26% comprehensive and 6% basic emergency obstetric care [EMOC] centers), 38% were partially functioning. Guidelines for the management of obstetric and newborn health complications were not available in all facilities. The poor technical quality of care was explained by a lack of skilled staff, equipment, and supplies [50].(Table 5).

**Table 5:** Synthesis of barriers to access RMNCAH services reported in the studies

Barriers to access MNCH healthcare services	Reproductive health services (family planning)	Maternal Health services (ANC, facility-based deliveries, post-natal care)	Newborn & children health care services
Geographic (distance, transport, roads)		+++	
Financial		+++	
Gender norms	+++	++	
Insecurity		+++	
Social-cultural norms		++	
Lack of knowledge of maternal dangers signs, newborn and children ‘s diseases		++	+
Perception of needs & benefits of using MNCH services		+	+
Perception of the health system (quality of care)		+++	

+reported in one study, ++ reported in two studies, +++ reported in more than two studies

#### Leadership and governance

##### Weak Ministry of Health leadership

Although the priorities for RMNCAH are defined by the MoH, the development partners (the WB, UNFPA, WHO, and UNICEF) orient the policies and programs that need to be implemented:

“*We sit together and plan, but they drive you to their own agenda*” (female, director, MoH, #4).

The UNFPA plays a key role in the development of key policies, strategic documents, and other guidelines for the delivery of reproductive health services. The MoH has limited capacity to manage the coordination of the different partners. This limited capacity has been attributed to the shortage of staff to support the directors in their day-to-day activities, including policy formulation, implementation, enforcement, and monitoring [44, 51, 52].

*“Normally, the directorate of RH (reproductive health) should have the capacity of 25 staff, but we are currently five: one for gender, gender/based violence; one for adolescents; two for safe motherhood; and one for family planning”* (female, director, MoH, #4).

*Less prioritization and integration of newborns’ and children’s health within reproductive health programs.*

Although the MoH has shown a strong political will to improve the health of pregnant women, adolescents, newborns, and children by drafting policies and health strategic plans, less attention has been given to children and newborns. According to policy documents, there is no technical working group for newborns’ and children’s health, while there is a technical working group for reproductive health [52].

At the MoH, there is no unit for child health. According to the participants, children and newborns’ health are included in the safe motherhood and nutrition programs. However, at the time of data collection, a health strategy plan focused on newborns was drafted. This document described the key strategies for newborn healthcare.

At the community level, one study reported weak integration of newborn care within the reproductive health program [39]. This study also indicated that community health workers (CHWs) faced challenges reaching all newborns for home visits since only 1 day was dedicated to newborn care [39].

#### Health workforce

##### Critical shortage of skilled healthcare workers

The most important challenge faced by South Sudan is the critical shortage of skilled healthcare workers. This shortage is also a challenge for managers at different levels of the health system. For instance, there are no technical program officers at the MoH to develop, coordinate, implement, and effectively monitor programs of primary health, preventive care, and reproductive healthcare [52].

The number of primary healthcare workers (clinical officers, midwives, and nurses) is insufficient to meet the basic needs of health and nutrition services. As a result, health facilities rely on traditional birth attendants (TBAs) and CHWs who do not have adequate skills to detect and manage major obstetric and newborn complications and to offer effective adolescents’ sexual and reproductive health services [42, 53]*.* According to a scoping review, community midwives who have been enrolled in the community midwife program to be posted in rural areas do not fully meet the international standard of skilled birth attendants due to the lack of a clear definition of and information about the birth attendant role. This review found only two technical reports on community midwifery programs in South Sudan [45]. Regarding the management of obstetric care, it was reported that health workers in health facilities do not conduct manual removal of retained placenta and do not assist in vaginal deliveries because of their lack of skills. This increases the burden on the referral system [50, 52].

Additionally, the numbers of trainers and training institutions are limited. There are a total of 24 training institutions for the whole country. Therefore, the MoH depends on expatriates and relies on neighboring health institutions to train their personnel [51].

At the community level, the number of CHWs is limited in the scope of their activities [39].

*“For the Boma Health Initiative, we do not have enough home health promoters to reach all the households. We have 2540 Bomas for the whole country, and we need three home health promoters in each Boma”* (male, director, MoH, #3).

##### Low motivation

Only two studies reported data on healthcare providers’ motivation [38, 53]. A qualitative study conducted in Juba with 18 healthcare providers reported poor supervision, a lack of training opportunities, and low salary to be the determinants influencing the motivation of healthcare providers and thus affecting their performance [53]. At the community level, two studies reported that a lack of incentives contributes to demotivating CHWs and has a negative impact on strategies for the deployment of community midwives in rural areas [39, 45].

#### Health information system

##### Incomplete and inconsistent information

According to all participants, *“the health information system is weak”.* Thus, it is difficult to plan and manage activities. For instance, one participant reported,

*“It is difficult to quantify the drugs needed by health facility, difficult to do the dataset. We can’t plan the health of a population if the health information system does not collect the data that we need for decision making”* (male, technical officer, implementing partner, #2).

Additionally, the current health system does not capture information at the community level.

*“We do not know how many children are using community-based interventions because data at the community level are not captured”* (male, technical officer, implementing partner #2).

A study reported that information on newborns from delivery registries is incomplete and that newborn admission is not well documented in health facilities managed by TBAs [39].

##### Lack of indicators of adolescents’ sexual reproductive health and gender-based violence

For adolescents, the policy document recommends the incorporation of indicators related to sexual and reproductive health into the health management information system and the disaggregation of the data according to gender and age [52, 54].

One of the important challenges faced by the MoH is quantifying the magnitude of the prevalence of gender-based violence. Gender-based violence has been reported to be a widespread issue, but the real magnitude of the problem is not well known because of the lack of data at the household level [39, 55]. Currently, the information is available only in reports at the health facility level. The next health management information system (HMIS) will capture data on adolescents and on gender-based violence:

*“In the new DHS that will come up next year (2020), the data will be disaggregated, and data on gender-based violence will be captured”* (female, director, MoH, #4).

#### Medicines and supplies

##### Lack of essential medicines

All documents reported a lack of essential medicines in health facilities [27, 33, 36, 39]. This is one of the greatest challenges in South Sudan. The MoH is in charge of pharmaceutical supply to all primary healthcare facilities, including those handled by NGOs. Pharmaceutical supply is based on a push system (focused on forecasting rather than demand). This system was described as being unresponsive to needs.

*“You could get anti-leishmaniasis [medication] coming to Western Equatoria where there is no leishmaniasis”* [44]*.*

The suspension of oil production prevented the MoH from taking over medicine supply in 2012 [44]. The international donors provided for a one-year emergency medicine fund (EMF). However, the drugs reached the country only in June 2014. Additionally, drugs from the EMF were not supplied to the three conflict-affected states according to a 2015 study [44].

*“There are no drugs that are being sent right now to Unity, Jonglei, and Upper Nile, because they say it’s too unstable”* [44]*.*

A study on newborn care reported that reproductive health kits did not provide 41 of 51 recommended newborn supplies for primary healthcare facilities [39]. In the same study, a health facility assessment conducted on a monthly basis indicated that “*nine newborn supplies were unavailable in each given month varying in the type of supply that was unavailable*” [39]. At the community level, no supplies were provided [39].

A nationwide facility survey conducted by the MoH reported that 40% of health facilities had all necessary drugs for the integrated management of childhood illnesses (IMCI) (amoxicillin, oral rehydration salts, and ciprofloxacin); however, each individual medicine was available in between 54 and 89% of facilities, only 60% of facilities had all drugs for antenatal care (SP/Fansidar, iron and folic acid), and only 50% of facilitates had all required vaccines in stock [56].

The national EMOC survey indicated that the essential medicine list to manage obstetric care was found to be inadequate and that there were frequent stock-outs of medicines on the list. The frequent stock-outs were explained by transportation delays (62%), administrative difficulties (16%), financial problems (8%), and stock-outs at the central store (6%) [50].

In the interviews, the participants mentioned frequent stock-outs of essential drugs, especially during the rainy season:

*“There are stock-outs, and some parts of the country are flooded almost two times per year, Great Upper Nile and Bahr Elghazal. If they do not stock up on the drugs during the dry seasons, they won’t be able to get drugs for almost six months”* (male, director, MoH #5).

In the peer-reviewed publications, qualitative studies reported the lack of medicines as a major contributor to the poor quality of care perceived by the population:

*“Most of the time, there is not enough medicine, and after long waiting times, we are asked to come back the next day. It is hard for me since I am unwell and too weak to do that. If I had money, I would have bought these medications from the private pharmacy”.*

(female participant) [43]*.*

The survey also reported the absence of clinical guidelines, especially for newborn care, preterm newborns, and sick newborns [50].

##### Poor infrastructure

All documents reported the poor infrastructure and equipment of healthcare facilities [50–52, 56, 57]. The national health facility assessment conducted by the MoH based on the analysis of 119 PHCCs and 118 PHCUs indicated that only 9% had the minimum required infrastructure (including a functioning ambulance), 6% had all essential equipment needed to perform IMCI consultations, and 67% had a working vaccine refrigerator [56].

Peer-reviewed articles reported that poor equipment at health facilities is a deterrent to the use of maternal health services and negatively affects the perception of quality of care:

*“In my understanding, the government facility is not well equipped for pregnant women to follow-up there. Also, women will not receive adequate services they are expecting and know the sex of the baby since they do not have ultrasound. In case of complications, this is a real problem since a specialist is not available, and trained TBAs or midwifery practice are very limited”* (male participant) [43]*.*

#### Health system financing

##### Low government funding and the challenges to access it

The government is able to dedicate only approximately 2–3% of its total annual budget to healthcare (< 4.5% GDP compared to the recommended 15%) [52]. The funding situation has drastically worsened due to general economic hardships experienced since 2015 [52]. According to the participants, the percentage of the total budget allocated to health was 1.9%, and this year, it increased by 2.4% due to lobbying:

*“The highest domestic allocation that the MoH has ever had was 7% of the national budget; that was in 2012, but this allocation has been declining from there each year until last year, it comes to 2%. And this year it is also 2%, but the challenge is that they do allocate, but when it comes to getting the funds, it becomes very difficult. Each time you request from the Ministry of Finance, they say the funds are not there. Last year, we were able to access only 30% of the total budget allocated. They allocate, but the physical money, they do not give it”* (male, director, MoH #5*).*

The allocation is made for the entire ministry; there are not separate budgets for each unit. As a result, none of the planned activities can be implemented, which increases dependency on donors:

*“We can’t do all these activities that are planned. We depend on partners. If the partners are funding 14 facilities to do some training, we can’t go beyond”* (female, staff, MoH, #2). *“Donors make us run here. We can’t depend only on donors; we need to be able to put something in the basket”* (male, director, MoH, #5).

##### International donors

Since 2012, the United Kingdom, Canada, the European Union, Sweden, and the United States have been the main international donors supporting the health system based on a contracting approach. According to a qualitative study, participants considered this mechanism “the only way to finance the health services compared to previous short-term humanitarian approaches”. However, some concerns were raised about this mechanism. It cannot address the substantial lack of capacity, and it fails to respond to emergencies [44].

##### Competing priorities for external funds

External funding for RMNCAH services is mainly provided by international donors. According to the policy and peer-reviewed documents, newborn and adolescent health receives less attention than maternal health and gender-based violence [42, 45, 52]. Therefore, the government is encouraging external funding to be rerouted to fund priority areas in reproductive health [54].

*“This year (2018), UNFPA has given to the MoH 124 409 US$ to support some activities based on the ASRH strategic plan (2018-2022), and in 2017, WHO supported some training on youth friendly reproductive health services for health providers in two states”* (female, director, MoH, # 4).

##### Lack of informed financial planning and budgeting

According to the policy documents, the MoH has challenges in tracking, debating, and lobbying for financial allocations [45, 52] (Table 6).

**Table 6:** Synthesis of the key health system gaps and their solutions for RMNACH programing

Health system gaps	Sub category	Solutions
Health financing	Low government funding and the challenges to access it	- Increase budget allocation and financial aid
	Competing priorities for external funds International donors	- Re streamline funds and better align them with the MOH’s priorities - Increase MoH stewardships
	Lack of informed financial planning and budgeting	- Improvement of planning budget
Health workforce	Critical shortage of skilled healthcare workers Low motivation	- Improve the capacity of institutions to increase intake and range of health professionals - Recruitment of qualified staff from within and diaspora, or from neighboring countries - Provide contingency recruitment plan/budget annually - Provide financial/ non -financial incentives
Medicines & supplies	Lack of essential medicines	- Increase the budget allocation
	Poor infrastructure	- Increase the budget allocation and advocate for low level government to fund its infrastructure
Leadership & governance	Less prioritization and integration of newborn’s and children’s health within RH programs Weak Ministry of Health leadership	- Creation of technical group for newborn and child health
		- Improve the capacity of the staff at the MOH - Development of a policy framework that allows leadership to direct, delegate, monitor and control health action. Empowered governance (oversight) committees and boards that support management functions at all levels of the health system.
Service delivery	Low coverage of RMNCAH services Barriers to accessing RMNCAH services Perception of poor quality of care	- Build, renovate, rehabilitation of health facilities - Increase skill’s staff through training, in job training - Increase drugs and equipment availability
Health system information	Incomplete and inconsistent information	- Strengthened facility and community- based surveillance and information system
	Lack of indicators of ASRH/GBV	- Incorporate ASRH indicators - Desegregate data (age/ sex)

## Discussion

Eight years have passed since South Sudan gained its independence, and progress towards the implementation of basic curative and preventive health services and subsequent policies remain slow. The 2012 suspension of oil production, ongoing conflict, the low capacity for improved stewardship by the MoH, the lack of accountability, and the low human resource capacity at the MoH are upstream factors that could explain the slow progress towards the implementation of the basic package of health services.

In a bid to negotiate a better deal with Sudan, South Sudan had to suspend oil production and export in January 2012. Oil represents approximately 98% of the country’s revenue. The suspension of oil production led to a loss of revenue of approximately $650 million each month and had a catastrophic impact on the health sector [44, 58]. This decision to suspend oil production has been vividly criticized by the international community [19, 58].

Only one year after the suspension of oil production in December 2013 until July 2016, ongoing armed conflicts between the government and the opposition occurred in Juba, the capital, that rapidly spread to the rest of the country [59]. The July 2016 conflict led to a disruption of health services in Upper Nile and Jonglei, where the health system is funded by the WB. In Juba, many international organizations evacuated their staff [20].

South Sudan remains one of the most volatile states in the world, and the ongoing peace process within the country remains fragile [60]. In December 2017, an attempt to arrange peace was unsuccessful. Continuous ethnic tensions between different political groups have continued to seriously impede the establishment of sustainable and real peace in the country [59].

This ongoing violence has critical economic and social impacts. As shown in our results, the budget for health has decreased drastically, and a high level of inflation is affecting the population. In January 2018, the Integrated Food Security Phase Classification reported that 48% of the population of South Sudan was facing acute food insecurity [61]. This economic crisis has also put more pressure on programs funded by donors. According to a 2018 report assessing the contribution of the HPF to strengthening the health system, the United Nations agencies’ humanitarian response plan of 1.7 billion USD for South Sudan was less than a quarter funded. All donors reported challenges with funding [20].

Medical supplies and the salaries of health staff have been the most affected building blocks because the government is in charge of both and has limited capacity to fund them [20, 44, 58]. Thus, the health system continues to depend heavily on external funding from international organizations and foreign governments. While the international donors have concerns regarding the level of fiduciary obligation and the lack of accountability within the MoH, they have continued to support the health system [44, 58, 62]. In February 2019, the WB approved another grant of 105.4 million USD to strengthen the health system [63]. This external funding is important but further jeopardizes the ability of the MoH to offer independent governance and stewardship. As shown by our results, the UN agencies and the HPF partners remain the main drivers of the policy process. This result aligns with the findings of a study published 9 years ago [64].

Several reports have indicated that governance in South Sudan is either absent or obstructed by the low capacity of government [58, 64]. However, recently, there have been some slight improvements in governance observed at the lower level of the system, especially at the county level [20, 65]. A 2018 report indicated that while no improvement has been observed at the national level, some progress has been noticed at the county level [20]. Another recent study assessing Swiss Red Cross programs to improve primary healthcare services reported an improvement in community participation at the county level through the implementation of needs-based programs [65].

Case studies from other post-conflict settings, such as Rwanda, Liberia, and Afghanistan, have shown that MoH leadership and the implementation of governance and accountability mechanisms are key determinants to strengthen health systems, increase the coverage of utilization of health services, and improve maternal and child health outcomes [66–68].

In relation to governance, the lack of capacity remains an important challenge to implementing basic health services and government policies. This result is in line with several reports and studies [20, 44, 58, 65]. According to a 2013 report from the Center of International Development of Harvard University, billions of dollars have been spent to “build capacities”, but no change has been observed. Some authors have stated that South Sudan is mired in a “capability trap” [19]. This concept entails two ideas: the importation of standardized practices to predetermined problems and the mismatch between the expectations and actual capacity of the government to implement even the most basic services. Donor agencies are currently using imported mechanisms and approaches, which reflects the concept of the capability trap and might explain the current failure to build the capacities of the government in South Sudan [19].

### Practical implications and research gaps

Strengthening South Sudan’s health system should be done through innovative and challenging approaches to building MoH capacities, implementing governance and accountability mechanisms, and increasing the national budget for the MoH.

While service delivery has been well documented in the literature, there are very large gaps in knowledge on the five other health system building blocks (leadership governance, health financing, health workforce, medicines and supplies, and health system information). Finally, more research is needed to document the “soft” dimensions of the health system, such as leadership, governance, accountability, and trust, between international donors and the Government of South Sudan and between the Government of South Sudan and its population.

### Strengths and limitations

Based on a systematic review of the literature, this study has analyzed the limited information on RMNCAH programs and policies in South Sudan. It provides a description of the policy landscape for the RMNCAH of the youngest nation in the world. This study also adds to the body of evidence on the key bottlenecks of the health system impeding the implementation of RMNCAH programs and policies. To our knowledge, this is the first study to attempt to understand the six health system building blocks and how they influence the implementation of RMNCAH policies in a post-conflict setting.

However, this study has some limitations. There were limited peer-reviewed publications exploring the health system building blocks (except for service delivery). Therefore, due to the quality of the evidence, it should be interpreted with caution. The limited number of interviews conducted [*n* = 8) was another limitation. More in-depth interviews with health manager staff from the MoH and international partners would have enriched the study. In addition, due to limited resources, we conducted interviews only at the national MoH. We were not able to interview staff from lower levels of the health system. Furthermore, due to the instability of the country and the multiple international partner dynamics, the policy context changes quickly; some of the findings may no longer be accurate at the time of the publication of this study. This is a unique case study. Thus, the transferability of the findings to other conflict-affected countries is likely to be limited.

## Conclusion

The 2012 suspension of oil production, ongoing conflict, the low capacity for improved stewardship by the MoH, the lack of accountability, and the low human resource capacity at the MoH are upstream factors that could explain the gaps in the health system and the slow progress towards the implementation of RMNCAH policies. The implementation of RMNCAH policies should be accomplished through innovative and challenging approaches to building the capacities of the MoH, establishing governance and accountability mechanisms, and increasing the health budget of the national government.

## Data Availability

The datasets used and/or analyzed in the current study are available from the corresponding author upon reasonable request.

## Abbreviations

BHI: Boma Health Initiative
BPHNS: Basic Package of Health and Nutrition Services
CH: County health
CHW: Community health worker
CMOC: Comprehensive obstetric care
DHS: Demographic Health Survey
DPT: Diphtheria, pertussis, tetanus
EMF: Emergency medicine fund
EMOC: Emergency obstetric care
EPI: Extended program for immunization
HMIS: Health management information system
HPF: Health Pooled Fund
IMCI: Integrated management child health
MoH: Ministry of Health
NGO: Nongovernmental organization
PHC: Primary healthcare
PHCU: Primary healthcare unit
RH: Reproductive health
RMNACH: Reproductive, maternal, newborn, adolescent, and child health
SH: State hospital
TBA: Traditional birth attendant
TH: Teaching hospital
UN: United Nations
WB: World Bank
WHO: World Health Organization
